# Oseltamivir-Resistant Influenza A(H1N1)pdm09 Viruses, United States, 2013–14

**DOI:** 10.3201/eid2101.141006

**Published:** 2015-01

**Authors:** Margaret Okomo-Adhiambo, Alicia M. Fry, Su Su, Ha T. Nguyen, Anwar Abd Elal, Elizabeth Negron, Julie Hand, Rebecca J. Garten, John Barnes, Xu Xiyan, Julie M. Villanueva, Larisa V. Gubareva, 2013–14 US Influenza Antiviral Working Group

**Affiliations:** Centers for Disease Control and Prevention, Atlanta, Georgia, USA (M. Okomo-Adhiambo, A.M. Fry, S. Su, H.T. Nguyen, A.A Elal, R.J. Garten, J. Barnes, X. Xiyan, J.M Villanueva, L.V. Gubareva);; Atlanta Research and Education Foundation, Atlanta (S. Su);; Battelle Memorial Institute, Atlanta (H.T. Nguyen, A.A. Elal);; Pennsylvania Department of Health, Harrisburg, Pennsylvania, USA (E. Negron);; Louisiana Office of Public Health, New Orleans, Louisiana, USA (J. Hand)

**Keywords:** influenza, influenza virus, viruses, influenza A(H1N1)pdm09 viruses, neuraminidase, neuraminidase inhibition, oseltamivir, zanamivir, resistance, pyrosequencing, H275Y mutation, United States

## Abstract

We report characteristics of oseltamivir-resistant influenza A(H1N1)pdm09 viruses and patients infected with these viruses in the United States. During 2013–14, fifty-nine (1.2%) of 4,968 analyzed US influenza A(H1N1)pdm09 viruses had the H275Y oseltamivir resistance–conferring neuraminidase substitution. Our results emphasize the need for local surveillance for neuraminidase inhibitor susceptibility among circulating influenza viruses.

During the 2013–14 influenza season, influenza A(H1N1)pdm09 virus was the predominant circulating virus (≈80%) in the United States for the first time since the 2009 pandemic (*1*). We report and describe characteristics of oseltamivir-resistant influenza A(H1N1)pdm09 viruses and patients infected with these viruses in the United States. 

## The Study

We requested that all US state public health laboratories submit influenza-positive specimens for virologic surveillance, including antiviral susceptibility testing, as described (*2*). In brief, every 2 weeks each laboratory was asked to send ≤5 specimens for all virus types for virus isolation and neuraminidase (NA) inhibition assay for oseltamivir, zanamivir, and, in a subset, laninamivir and peramivir (*3*). All oseltamivir-resistant viruses were tested for the H275Y substitution in NA by pyrosequencing (*4*). Unpropagated influenza A(H1N1)pdm09 virus–positive clinical specimens were screened for the H275Y substitution by pyrosequencing (Technical Appendix). If a cluster (>2 viruses) of oseltamivir-resistant A(H1N1)pdm09 viruses was detected, the state was asked to submit additional influenza A(H1N1)pdm09 virus specimens for testing.

We attempted to collect information by using a standard form from all patients with oseltamivir-resistant virus infection and from a sample of patients with oseltamivir-susceptible virus infection. A 2:1 (susceptible:resistant) sample was randomly selected from the list of tested specimens from the same age group in each state (<5, 5–17, 18–64, and ≥65 years). Patients with oseltamivir-resistant or -susceptible virus infections were compared by using conditional logistic regression models that controlled for age. Full NA and hemagglutinin sequence analysis was performed on all resistant viruses and a subset of susceptible viruses.

**Figure 1:**
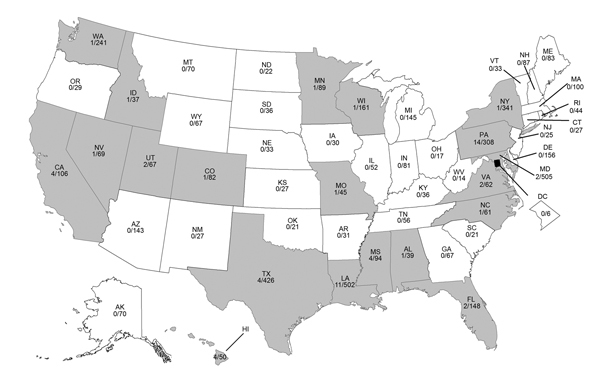
Geographic distribution of oseltamivir-resistant influenza A(H1N1)pdm09 viruses, United States, 2013–14. Gray indicates the presence of an oseltamivir-resistant virus. Number of oseltamivir-resistant A(H1N1pdm)09 viruses divided by total number of viruses tested is shown for each state. Oseltamivir-resistant A(H1N1)pdm09 viruses were significantly more prevalent in Louisiana (LA) (p = 0.04, by Fischer 2-sided exact test), Pennsylvania (PA) (p<0.001), Mississippi (MS) (p = 0.02), Hawaii (HI) (p = 0.02), and California (CA) (p = 0.03) than in all other states combined.

During October 1, 2013–April 30, 2014, a total of 4,968 influenza A(H1N1)pdm09 virus specimens collected from 50 US states and 2 territories were tested for antiviral susceptibility (1,811 virus isolates and 3,157 clinical specimens). A total of 59 (1.2%) influenza A(H1N1)pdm09 viruses from 20 states had the H275Y NA substitution conferring resistance to oseltamivir and peramivir (Figure 1; Table 1). None of 1,811 virus isolates was resistant to zanamivir.

**Table 1:** Neuraminidase inhibitor susceptibility for influenza A(H1N1)pdm09 viruses, United States, October 1, 2013–April 30, 2014*

Method of testing	Neuraminidase inhibitor			
	Oseltamivir	Zanamivir	Peramivir	Laninamivir
Neuraminidase inhibition assay†				
No. virus isolates tested‡	1,811	1,811	1,431	352
No. oseltamivir susceptible (mean IC_50_ ± SD, nmol/L)	1,792 (0.19 ± 0.14)	1,811 (0.18 ± 0.06)	1,412 (0.06 ± 0.02)	352 (0.23 ± 0.08)
No. oseltamivir resistant, (mean IC_50_±SD, nmol/L)	19 (181.31 ± 67.63)	0	19 (17.71 ± 6.83)	0
Resistance, %	1.1	0	1.3	0
Pyrosequencing§				
No. clinical specimens tested	3,157	NA	3,157	NA
No. H275 wild-type	3,117	NA	3,117	NA
No. H275 variants	40	NA	40	NA
Resistance, %	1.3	NA	1.3	NA
Total				
No. virus tested	4,968	1,11	4,588	352
No. resistant viruses	59	0	59	0
Resistance, %	1.2	0	1.3	0

*IC_50_, 50% inhibitory concentration; NA, not applicable. †H275Y confirmed in virus isolate by pyrosequencing and full neuraminidase sequencing. ‡Most (99.8%) influenza A(H1N1)pdm09 virus isolates were characterized as A/California/7/2009-like, the influenza A (H1N1) component of the 2013–2014 Northern Hemisphere influenza vaccine. §Includes pyrosequencing data from New York contract laboratory and data submitted by 19 state public health laboratories in Arizona, California, Colorado, Delaware, Florida, Georgia, Hawaii, Idaho, Maine, Maryland, Massachusetts, Michigan, Minnesota, New York, Pennsylvania, Texas, Utah, Washington, and Wisconsin.

Viruses with the H275Y substitution were detected in patient specimens collected during October 7, 2013–March 25, 2014; monthly prevalence ranged from 0.8% to 2.5%. Among 49 (83.0%) patients with a resistant virus infection and available information, 15 (30.6%) received oseltamivir before specimen collection (Table 2). Prior oseltamivir use was more frequent among hospitalized patients and patients with resistant virus infections than those with susceptible virus infections. Among those with prior exposure, 6 (40.0%) patients with oseltamivir-resistant and none with oseltamivir-susceptible virus infections were immunocompromised (p = 0.03). No differences were found between patients with oseltamivir-resistant or -susceptible virus infections.

**Table 2:** Characteristics of patients infected with oseltamivir-resistant and -susceptible A(H1N1)pdm09 viruses, United States, October 1, 2013–April 30, 2014*

Characteristic	Patients with oseltamivir-resistant infections, n = 49†	Patients with oseltamivir-susceptible infections, n = 93†	p value	OR (95% CI)
Median age, y (IQR)	25 (14–53)	24 (18–46)	0.86	NA
Male sex	22/49 (45)	37/92 (40)	0.59	1.21 (0.59–2.46)
White race	32/47 (68)	64/88 (73)	0.84	0.73 (0.37–1.89)
Exposure to oseltamivir before specimen collection	15/49 (31)	9/93 (10)	0.002	4.12 (1.65–10.31)
Outpatients	2/32 (6.3)	1/65 (1.5)	0.21	4.27 (0.37–48.9)
Hospitalized patients	13/17 (76)	8/27 (30)	0.003	7.72 (1.92–31.06)
Exposure to others in household receiving antiviral drugs before patient’s illness	5/33 (15)	4/62 (6)	0.16	2.64 (0.63–11.07)
Any underlying medical conditions	25/49 (51)	50/93 (54)	0.8	0.85 (0.39–1.86)
Chronic pulmonary disease	5/49 (10)	7/93 (8)	0.59	1.2 (0.28–5.07)
Chronic cardiac disease	6/49 (12)	10/93 (11)	0.79	1.38 (0.39–4.83)
Diabetes mellitus	6/49 (12)	8/93 (9)	0.49	1.82 (0.53–6.26)
Immunosuppressive conditions‡	8/49 (16)	6/93 (7)	0.07	3.2 (0.1–10.3)
Other§	9/49 (18)	23/93 (25)	0.39	0.66 (0.27–1.61)
Hospitalized during influenza illness	17/49 (35)	27/92 (29)	0.51	1.51 (0.67–3.43)
Patient died	7/47 (15)	6/93 (6)	0.1	2.8 (0.86–9.14)
Others in the household were ill before patient’s illness	12/32 (38)	21/65 (32)	0.61	1.2 (0.47–3.05)

*OR, odds ratio adjusted for age group; IQR, interquartile range; NA, not applicable. †Values are no./total (%) unless otherwise indicated. ‡Long history of steroids treatment, HIV/AIDS, solid organ transplant, lupus, solid tumor malignancy, hypothyroidism, leukemia, and pituitary condition. §Morbid obesity, chronic liver disease, neurologic disorders, chronic kidney disease, seizure, epilepsy, and depression.

Most resistant viruses were clustered in 5 states (California, Hawaii, Louisiana, Mississippi, and Pennsylvania) (Figure 1). Among patients with oseltamivir-resistant virus infection, only 1/4 from California, 0/4 from Hawaii, 3/11 from Louisiana, 1/3 from Mississippi, and 0/14 from Pennsylvania had exposure to oseltamivir before specimen collection. All patients from Pennsylvania except 1 attended 1 of 2 universities (among 7 participating students, none shared classes, residences, or social events). There were no epidemiologic links between other patients. Limited information was available for oseltamivir-treated patients with resistant and susceptible virus infections (Technical Appendix Table).

**Figure 2:**
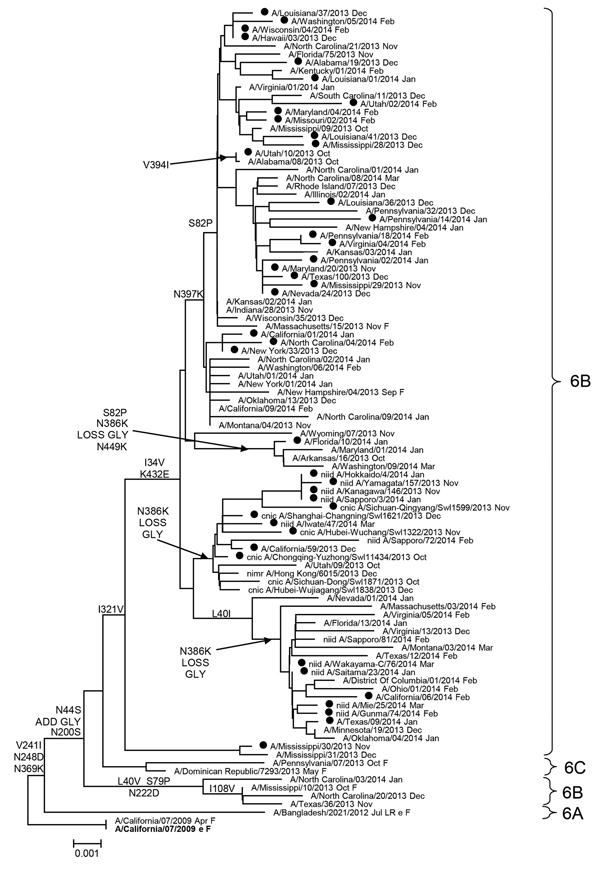
Evolutionary relationships among influenza A (H1N1)pdm09 virus neuraminidase genes, United States, 2013–14. Phylogenetic tree was generated by using MEGA software v5.2 (http://www.megasoftware.net/) and the neighbor-joining method. Evolutionary distances were computed by using the maximum composite likelihood model. Analysis included 100 representative A(H1N1)pdm09 neuraminidase gene sequences. Scale bar indicates nucleotide substitutions per site. Solid circles indicate oseltamivir-resistant H275Y markers. A/California/07/2009 (current Northern Hemisphere vaccine strain) virus was used as a reference for ancestry (root) and numbering. F, Centers for Disease Control and Prevention reference antigen; Oct, October 2013; Nov, November 2013; Dec, December 2013; Jan, January 2014; Feb, February 2014; GLY, glycosylation.

Most hemagglutinin sequences from US influenza A(H1N1)pdm09 viruses collected since October 1, 2013, belonged to the 6B genetic group, and there was minimal separate clustering between susceptible and resistant viruses (Technical Appendix Figure 1). Similar results were observed for the phylogenetic tree of the NA gene (Figure 2). NA sequences from resistant viruses in the United States with the H275Y substitution were generally scattered among other susceptible viruses from genetic group 6B viruses. Most (>99%) influenza A(H1N1)pdm09 viruses currently in circulation have NA substitutions V241I and N369K (Technical Appendix Figure 2). There was >1 cluster of NA sequences with the N386K substitution; each cluster contained susceptible and resistant viruses. Most (>89%) resistant viruses from the United States do not have the N386K mutation.

## Conclusions

During the 2013–14 influenza season, prevalence of oseltamivir-resistant influenza A(H1N1)pdm09 viruses was low (≈1%) in the United States, although prevalence was higher in a few states. Most patients infected with an oseltamivir-resistant influenza A(H1N1)pdm09 virus had no prior exposure to oseltamivir. These findings are consistent with a low, and locally variable, level of circulation of resistant viruses. In our study, exposure to oseltamivir before specimen collection was more common among hospitalized patients with resistant virus infections than those with susceptible virus infections. We cannot differentiate whether these viruses emerged during treatment or were present before treatment, but many patients were immunocompromised, a condition associated with emergence of resistance during treatment (*5*).

Before 2007, resistance to NA inhibitors among influenza viruses circulating globally was low (<1%) (*6*). However, the 2007–08 influenza showed an emergence of oseltamivir-resistant seasonal influenza A(H1N1) H275Y viruses at variable prevalence (*6*), and by the 2008–09 season, many countries were reporting up to 100% oseltamivir resistance (*7*). The sharp increase in seasonal influenza A(H1N1) H275Y viruses from <1% to ≈100% was not attributed to oseltamivir use (*8*), but was probably caused by evolutionary advantage of H275Y variants. Studies suggest that permissive NA mutations, including R222Q, V234M, and D334N, counteracted the detrimental effect of H275Y on NA function and virus replicative properties, thus enabling virus to remain fully functional (*9*). The exact mechanism(s) responsible for evolutionary advantage of seasonal influenza A(H1N1) H275Y viruses over oseltamivir-susceptible viruses remain unknown.

Since emergence of influenza A(H1N1)pdm09 virus in 2009, there is concern that the H275Y substitution may become fixed in the viral genome, as it did in seasonal influenza A(H1N1) virus in 2008–09. Oseltamivir-resistance among influenza A(H1N1)pdm09 viruses during their first 2 seasons in circulation (2009–11) remained low (<1%) (*2*,*5*). However, during June–August 2011, in Newcastle, New South Wales, Australia, a cluster of oseltamivir-resistant influenza A(H1N1)pdm09 H275Y viruses was detected among patients without prior oseltamivir exposure (*10*), suggesting community transmission. These H275Y viruses had permissive mutations, V241I and N369K, in addition to N386S (*10*), which was similar to H275Y viruses isolated in 2012 from Dutch travelers returning from Spain (*11*). These mutations were believed to offset the destabilizing effect of H275Y and possibly enhance virus transmissibility. The substitutions V241I, N369K, or N386S were not present in influenza A(H1N1)pdm09 virus when it emerged in 2009. However, since 2011, circulating influenza A(H1N1)pdm09 viruses have acquired these substitutions, coinciding with increasing evidence for community transmission of influenza A(H1N1)pdm09 H275Y viruses in the United States and other countries (*2*,*12*).

All influenza A(H1N1)pdm09 viruses circulating in the United States in 2013–14 had V241I and N369K substitutions, and ≈10% of resistant viruses and ≈20% of susceptible viruses had an additional substitution (N386K). All influenza A(H1N1)pdm09 H275Y viruses detected in China and Japan in 2013–14 had all 3 substitutions (*13*). In combination with the H275Y substitution, V241I or N369K enhances surface expression and activity of NA (*14*). The N386S substitution and the recently detected N386K substitution result in loss of a glycosylation site (*15*). Although the potential role of these changes in virus spread was suggested (*10*), no direct evidence is available. Close monitoring for the N386K/S substitution may provide information needed to delineate its role in virus spread. In addition to permissive NA mutations, other properties, such as antigenic novelty, which might provide an advantage to oseltamivir-resistant viruses and facilitate their spread, should also be monitored.

The potential for emergence and spread of oseltamivir-resistant influenza A(H1N1)pdm09 viruses, coupled with limited pharmaceutical options against influenza, emphasizes the need for local surveillance for NA inhibitor susceptibility among circulating influenza viruses. Studies on biologic characteristics (e.g., replication in and transmissibility from ferrets) of influenza A(H1N1)pdm09 virus community isolates with H275Y and other permissive mutations are ongoing.

Technical Appendix Supplementary methods for analysis of oseltamivir-resistant influenza A(H1N1)pdm09 viruses, United States, 2013–14.
