# Supplementary material for: Oseltamivir-Resistant Influenza A(H1N1)pdm09 Viruses, United States, 2013–14
Source: Emerg Infect Dis. 2015 Jan;21(1):136–41. doi: 10.3201/eid2101.141006 (PMC4285251; doi:10.3201/eid2101.141006)
Supplement: Technical Appendix — Supplementary methods for analysis of oseltamivir-resistant influenza A(H1N1)pdm09 viruses, United States, 2013–14. [file 14-1006-Techapp-s1.pdf]

Article DOI: http://dx.doi.org/10.3201/eid2101.141006

Oseltamivir-Resistant Influenza A(H1N1)pdm09 Viruses, United States, 2013–14

Technical Appendix

Supplemental Methods for Analysis of Oseltamivir-Resistant Influenza A(H1N1)pdm09 Viruses, United States, 2013–14

Neuraminidase inhibition (NI) assays (*1*) and pyrosequencing (*2*) were performed according to standard protocols of the Centers for Disease Control and Prevention (CDC) (Atlanta, GA, USA) (fluantiviral@cdc.gov). For national virologic surveillance, NI testing for most (80%) virus isolates (n = 1,811) was performed by public health laboratories in California, Wisconsin, and Utah that were contracted by the Association for Public Health Laboratories (Silver Spring, MD, USA). The remaining (20%) NI testing was performed by CDC. Results of the NI assay (50% inhibitory concentration) were interpreted and reported in accordance with recommendations of the Influenza Antiviral Working Group of the World Health Organization (*3*), in which influenza A viruses with <10-fold change in 50% inhibitory concentration are characterized as exhibiting normal inhibition by the respective neuraminidase inhibitor, and those with 10–100-fold and >100-fold change as exhibiting reduced and highly reduced inhibition, respectively. Viruses showing reduced and highly reduced inhibition are genetically analyzed to detect molecular markers of neuraminidase inhibitor resistance. In this study, virus isolates showing highly reduced inhibition by oseltamivir were tested by pyrosequencing at CDC to confirm the presence of the H275Y marker of resistance.

Of 3,157 clinical specimens tested primarily by pyrosequencing to detect the H275Y marker, 40% were tested for national surveillance by the Wadsworth Center, New York State Department of Health (Albany, NY, USA), which was also contracted by the Association for Public Health Laboratories, and 60% were tested for individual state surveillance by 19 other public health laboratories, who then shared their data with CDC. Further comprehensive genetic analysis was performed on drug-resistant viruses detected by NI assay or pyrosequencing. Antiviral susceptibility data from all above testing sources were consolidated for publication in the weekly CDC FluView report (*4*) on national virologic surveillance. When necessary, surveillance was enhanced by increasing

sampling and testing in specific regions exhibiting higher than the national frequency of neuraminidase inhibitor resistance.

References

<jrn>1. Okomo-Adhiambo M, Sleeman K, Lysen C, Nguyen HT, Xu X, Li Y, et al. Neuraminidase inhibitor susceptibility surveillance of influenza viruses circulating worldwide during the 2011 Southern Hemisphere season. Influenza Other Respir Viruses. 2013;7:645–58. PubMed http://dx.doi.org/10.1111/irv.12113</jrn>

<jrn>2. Deyde VM, Sheu TG, Trujillo AA, Okomo-Adhiambo M, Garten R, Klimov AT, et al. Detection of molecular markers of drug resistance in 2009 pandemic influenza A (H1N1) viruses by pyrosequencing. Antimicrob Agents Chemother. 2010;54:1102–10. PubMed http://dx.doi.org/10.1128/AAC.01417-09</jrn>

<jrn>3. Meetings of the WHO working group on surveillance of influenza antiviral susceptibility – Geneva, November 2011 and June 2012. Wkly Epidemiol Rec. 2012;87:369–74. PubMed</jrn>

<eref>4. Centers for Disease Control and Prevention. Seasonal influenza activity and surveillance [cited 2014 May 21]. http://www.cdc.gov/flu/weekly/</eref>

Technical Appendix Table. Comparison of oseltamivir-treated patients infected with oseltamivir-resistant and -susceptible influenza A(H1N1)pdm09 viruses, United States, October 1, 2013–April 30, 2014*

Characteristic	Patients with oseltamivir-resistant A (H1N1)pdm09 infections (n = 49)	Patients with oseltamivir-susceptible A (H1N1)pdm09 infections (n = 93)	p value
Treated with oseltamivir anytime during illness†	37/49 (76)	67/93 (72)	0.66
Documented full course of treatment†	22/37 (59)	40/67 (60)	0.98
	Full treatment (n = 22)	Full treatment (n = 40)	NA
Median age, y	36 (19–53)	25 (19–54)	0.29
Any underlying medical conditions	8/21 (38)	15/39 (38)	0.98
Days of influenza illness (all patients)	8 (6–20), (n = 15)	5 (4–7), (n = 33)	0.01
Hospitalized patients‡	23.5 (10–33), (n = 6)	6.5 (4.5–13.5), (n = 12)	0.05
Outpatients	7 (6–8), (n = 9)	5 (4–6), (n = 21)	0.05
Days of fever§	3 (0–7)	2 (2–3)	0.31
Hospitalized during influenza illness‡	12/22 (55)	19/40 (48)	0.60
Patient died	4/21 (19)	3/40 (8)	0.18
	Any documented treatment (n = 37)	Any documented treatment (n = 67)	NA
Days of influenza illness (all patients)	7 (5–10) (n = 22)	6 (5–10) (n = 46)	0.05
Hospitalized patients‡	20 (10–33) (n = 7)	6 (5–10) (n = 17)	0.03
Outpatients	7 (4–7) (n = 15)	5 (5–7) (n = 29)	0.39

*Values are no./total (%) or median IQR. Clinicians were unaware of surveillance test results. The small sample size and lack of complete information for all patients limits the conclusions that can be drawn from the information in this table. Analysis was not age adjusted because of sample size. Patients with missing data were excluded from the analysis. Sample sizes for variables with incomplete information are shown. NA, not applicable.
†Treatment information was collected by self-report only (n = 15), medical chart review (n = 82), and was missing for 7. Of 104 treated patients, 62 (60%) had documentation of a full course (5 d, 2×/d) of oseltamivir either by self-report or medical record.
‡Among hospitalized patients who received a full treatment course of oseltamivir, 4 patients with a resistant virus infection had an immunosuppressive condition compared 6 among hospitalized treated patients with susceptible virus infections (p = 0.88).
§No information on antipyretic use was collected.

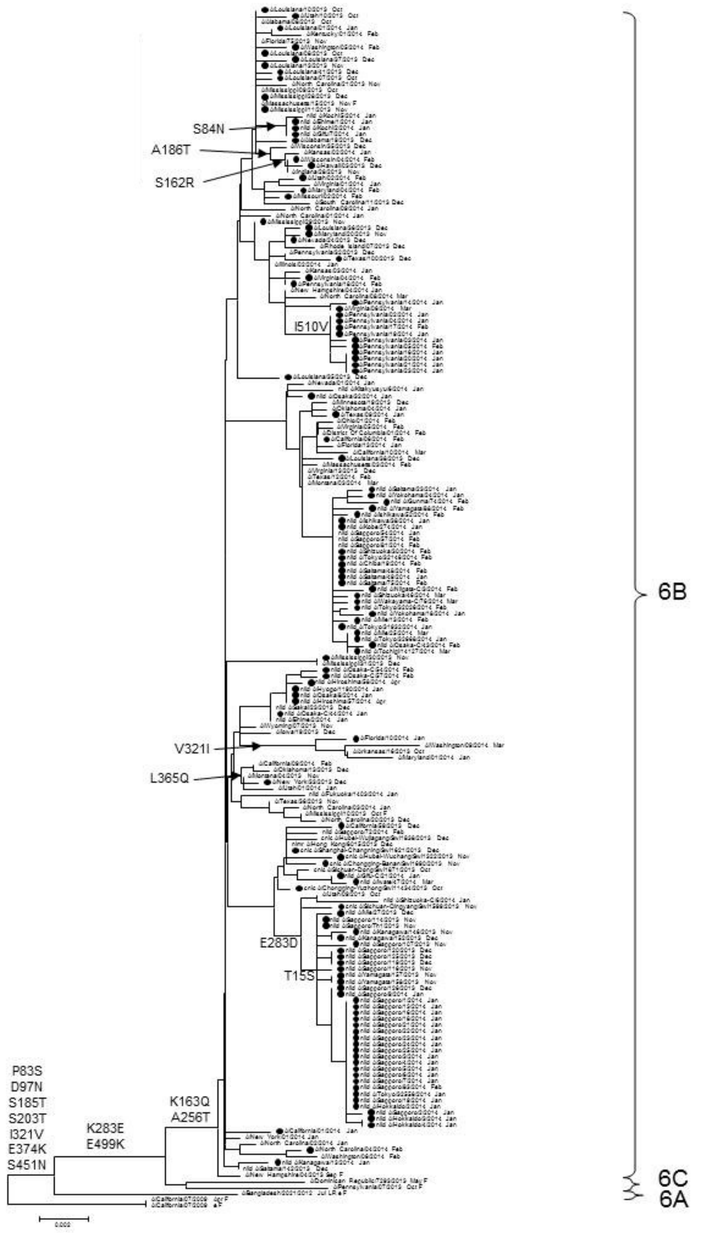

Technical Appendix Figure 1. Evolutionary relationships among influenza A (H1N1)pdm09 hemagglutinin (HA) genes, United States, 2013–14. Phylogenetic tree was generated by using the MEGA software package v5.2 (http://www.megasoftware.net/) and the neighbor-joining method. Evolutionary distances were computed by using the maximum composite likelihood model. Analysis included 193 representative A(H1N1)pdm09 HA gene sequences. Scale bar indicates nucleotide substitutions per site. Solid circles indicate oseltamivir-resistant H275Y markers. A/California/07/2009 (current Northern Hemisphere vaccine strain) virus was used as a reference for ancestry (root) and numbering. F, Centers for Disease Control and Prevention reference antigen; Oct, October 2013; Nov, November 2013; Dec, December 2013; Jan, January 2014; Feb, February 2014; GLY, glycosylation.

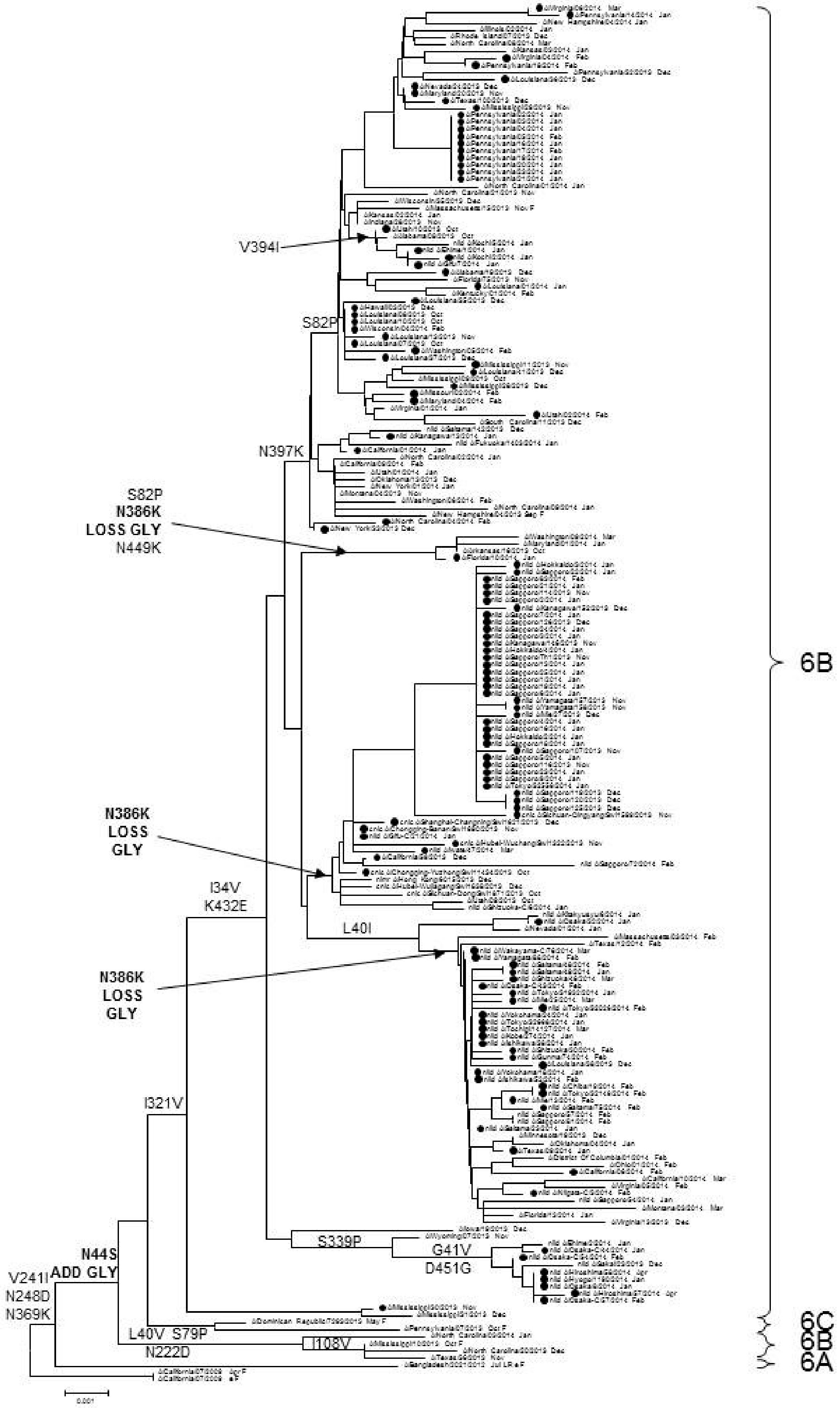
V394I
S82P
N397K
S82P
N386K
LOSS GLY
N449K
N386K
LOSS
GLY
I34V
K432E
L40I
N386K
LOSS
GLY
I321V
S339P
G41V
D451G
N44S
ADD GLY
V241I
N248D
N369K
L40V S79P
N222D
I108V
6B
6C
6B
6A

Technical Appendix Figure 2. Evolutionary relationships among influenza A (H1N1)pdm09 neuraminidase (NA) genes, United States, 2013–14. Phylogenetic tree was generated by using the MEGA software package v5.2 (http://www.megasoftware.net/) and the neighbor-joining method. Evolutionary distances were computed by using the maximum composite likelihood model. Analysis included 193 representative A(H1N1)pdm09 NA gene sequences. Scale bar indicates nucleotide substitutions per site. Solid circles indicate oseltamivir-resistant H275Y markers. A/California/07/2009 (current Northern Hemisphere vaccine strain) virus was used as a reference for ancestry (root) and numbering. F, Centers for Disease Control and Prevention reference antigen; Oct, October 2013; Nov, November 2013; Dec, December 2013; Jan, January 2014; Feb, February 2014; GLY, glycosylation.
